# Effects of Metformin on TSH Levels and Benign Nodular Goiter Volume in Patients Without Insulin Resistance or Iodine Insufficiency

**DOI:** 10.3389/fendo.2019.00465

**Published:** 2019-07-17

**Authors:** Patricia Borges dos Santos, Larissa Nascimento Gertrudes, Flavia Lucia Conceição, Bruno Moulin de Andrade, Denise Pires de Carvalho, Mario Vaisman, Patricia de Fatima dos Santos Teixeira

**Affiliations:** Post Graduation Program in Endocrinology, Medicine School, Federal University of Rio de Janeiro, Rio de Janeiro, Brazil

**Keywords:** benign thyroid nodule, thyroid nodules volume, thyroid volume, metformin, insulin resistance, TSH

## Abstract

**Objectives:** To evaluate the impact of metformin (MTF) use on TSH levels, thyroid volume and volume of benign thyroid nodules (TNs). Additionally, to study if iodine status influences the outcomes.

**Methods:** A total of 23 euthyroid patients (42 TNs) with benign thyroid nodules, diagnosed by fine needle aspiration biopsy, were randomly assigned to MTF or placebo (P) use for 6 months. Serum TSH, homeostatic model assessment for insulin resistance (HOMA-IR), and urinary iodine concentrations (UIC) were assessed. Ultrasound was used to evaluate TNs and thyroid volumes (TV) and their variations throughout the study. Diabetic patients, those undergoing levothyroxine replacement, and/or using thyroid- or insulin level-influencing drugs were excluded.

**Results:** The sample consisted predominantly of patients without IR. Both intervention groups were similar regarding several confounding variables and showed a comparable median UIC. Serum TSH decreased significantly after MTF (−0.21 vs. 0.09 mUI/L in the P group; *p* = 0.015). At 6 months, no significant variations were found between groups with respect to TN volumes, TV, HOMA-IR, or body mass index (BMI). However, a tendency toward enlargement of TV with placebo (16.0%; *p* = 0.09) and a protective effect of MTF on growing TN (OR: 0.25; CI 0.05–1.20) was detected after excluding patients with IR (a lower UIC subgroup). The reduction on TSH levels with MTF maintained in the population without iodine insufficiency (−0.24 vs. +0.07 in the P group; *p* = 0.046) and was accentuated in those with excessive or more than adequate UIC (−0.69; *p* = 0.043). A protective effect of MTF on growing TN was suggested (OR: 0.11; IC: 0.02–0.84) in those with higher UIC.

**Conclusions:** This study demonstrated that MTF caused a reduction in TSH levels in benign nodular goiter. This effect was more accentuated in patients with higher levels of UIC and was accompanied by a suggested protective effect on TN enlargement.

## Introduction

Thyroid nodules (TNs) are routinely found in worldwide clinical practices and are detected in up to 50 to 60% of subjects (1). However, even though fine needle aspiration biopsy (FNAB) confirms it's benign diagnosis in up to 75% of cases, a subgroup that might grow over time and sometimes requiring thyroidectomy (1). Identifying which type of benign TNs are at risk of developing this outcome and discovering the possibility of non-surgical strategies or drugs that could stop this evolution has been the subject of worldwide research.

Insulin is one of the hallmarks in the pathogenesis of thyroid growth since it stimulates thyrocyte proliferation similar to thyroid-stimulating hormone (TSH). It has been demonstrated that patients with insulin resistance (IR) and high serum levels of insulin might have large thyroid volumes and a higher prevalence of thyroid nodules (2, 3). An association between IR and differentiated thyroid carcinoma has already been demonstrated (4, 5).

Metformin (MTF), which improves peripheral insulin sensitivity and is commonly used to treat type 2 diabetes, has been studied with respect to decreasing thyroid volume and nodule size in subjects with IR (6–10) and might be a potential antitumor drug (11) The mechanism of its action can be attributed to its growth-inhibitory effect via insulin/insulin-like growth factor (IGF) signaling and AMP-activated protein kinase/mammalian target of rapamycin (AMPK/mTOR) pathway (11). A meta-analysis confirmed that MTF can also be associated with low levels of serum TSH in IR patients (12). The exact mechanism has not been completely elucidated, but it might involve inhibitory effects on the AMPK system in the hypothalamus (12).

Over recent years, various publications have focused on the effects of MTF on nodular volume and TSH levels in patients with IR (6–10, 12–19). However, there are insufficient data in the literature evaluating whether these effects can also be observed in patients without IR. Furthermore, no study has evaluated whether or not the presence of other goitrogenic factors influences, such as iodine insufficiency, the response to MTF on nodular goiter volume or serum TSH.

In the present study, we hypothesized that metformin use can exert some influence over enlargement of thyroid volume (TV) and of benign TNs over time and also reduces serum TSH levels. We evaluated the relationships between iodine status and metformin treatment on variations of TV and benign TN volumes.

## Subjects and Methods

Patients with previous diagnosis of benign thyroid nodule (BTN) disease, confirmed by FNAB, were recruited from the outpatient clinic of Clementino Fraga Filho University Hospital of Federal University of Rio de Janeiro (UFRJ). All patients were >18 years and gave their written, informed consent to the researcher. The protocol was approved by the Institutional Ethical Committee (CAAE:35585214.0.0000.5257), and the study was registered at ClinicalTrial.gov (NCT03183752). An interview was conducted to assess inclusion and exclusion criteria before the patient was selected for the study. At the time of this interview, a new ultrasound was performed to reevaluate the specific ultrasound pattern of BTN since only those showing solid or predominantly solid formations were included in the study.

The exclusion criteria consisted of several parameters: (1) pregnancy; (2) diabetes (diagnosed before or during the study); (3) acromegaly; (4) hepatic or cardiac insufficiency; (5) creatinine levels >1.4 mg/dL: and (6) any previous use of MTF. We also excluded patients who were receiving levothyroxine, corticoids, or any weight loss medication in the past 6 months. Previous radioiodine treatment was also considered an exclusion criterion, as was MTF intolerance to doses <1.0 g/day during follow-up.

After initial evaluation and baseline assessment of studied parameters, the patients were randomized into two intervention groups. Randomization was done by blocks, comprising four patients each, in a double-blinded manner. Similar tablets containing MTF or placebo were precisely counted and given to each patient. All patients were instructed to initially take 1 MTF tablet/day (500 mg/tablet); however, patients were instructed to take MTF with a weekly increment until reaching 3 tablets/day. All patients returned after 2 months for biochemical assessments (creatinine levels) and evaluation of adherence to the study protocol. A physician not blinded and not enrolled in the ultrasound and clinical/laboratory evaluations, was responsible for this assessment. A blinded physician performed a new ultrasound after 6 months of follow-up (the same one who had assessed baseline ultrasound pattern). However, this researcher was also blinded to the first ultrasonography parameters and also to the intervention group to which the patient was allocated.

Throughout the study, patients who were already on drugs (corticoids, weight loss medications, diabetes drugs), which could influence serum insulin levels, were excluded. Patients not adhering to the filled criteria of the protocol were also excluded. No adherence to the protocol was assumed when there was a return of >20% of the total tablets for the period.

All participants underwent thyroid ultrasonography, specific anamnesis, and clinical and laboratory assessment in the first evaluation and after 6 months of follow-up.

### Anthropometric Measurements

Participants were weighed and measured without shoes or cap. Waist circumference (WC) was measured with a folding tape at natural waistline (the level of the umbilical). Body mass index (BMI) was calculated by means of the following formula: BMI = weight/height^2^.

### Thyroid Morphology

All ultrasonographic evaluations were performed by the same physician, using a high-frequency SIEMENS-AUSONX X300 multifrequency transducer (12 MHz). TV and TN volume were calculated by the formula: length × width × thickness ×0.52 of each lobe and the isthmus. A TN was considered to have growth if it showed an increase in its volume throughout the time by >10%. Additionally, a reduction of >10% was necessary to assume that a TN had shrunk. Variations in TV were also considered only when occurred by >10%.

### Laboratory Assessments

Serum samples were obtained by venipuncture after 8 h fasting and included TSH, glucose, insulin for HOMA-IR calculation, lipid profile and thyroid peroxidase antibody (TPO-Ab).

Serum levels of TSH and anti-thyroid peroxidase antibodies (TPO-Ab) were determined by immunochemiluminesce (Immulite, Diagnostic Products, Los Angeles, CA). Reference ranges for TSH were 0.4–4.0 μUI/mL, and TPO-Ab >35 UI/mL was considered positive. The intra-assay coefficients of variation were 3.8–12.5% and 4.3–5.6% for TSH and TPO-Ab, respectively while the inter-assay coefficients of variation were 4.6–12.5% and 7.8–10.5%, respectively. Glucose was assayed by hexoquinase (ADVIA Chemistry, Siemens). The reference range for glucose was based on the guidelines of the American Diabetes Association (ADA) and the Brazilian Diabetes Society (SBD). Insulin levels were measured using the Chemiluminescence Imunoassay Kit (ADVIA Centauro, Siemens) with reference ranges 3.0–25 μU/ml. The intra- and inter-assay coefficients of variation were 3.2–4.6% and 2.6–5.9%, respectively. HOMA-IR was calculated with the formula (Fasting plasma insulin [IU/ml] × fasting plasma glucose [nmol/L])/22.5. IR was considered when HOMA-IR >2.7 (20).

Spot urine samples were obtained from each participant for assessing urinary iodine concentration (UIC) that was determined by Inductively Coupled Plasma Mass Spectrometry (ICP-MS-Spectroquant^®^ Iodine Test–Merch KGaA, Germany). The manufacturer's reference range was 26 to 705 μg/L. The classification of adequate iodine status was made according to the World Health Organization (WHO) for adults (100 to 199 mcg/L) (21).

### Statistical Analysis

SPSS software (version 21.0 for windows; SPSS Inc., Chicago, IL, USA) was used for statistical analysis. Continuous variables were expressed as medians (interquartile range [IQ]) or median (minimum–maximum values) and compared between two independent groups using the Mann-Whitney test. Categorical variables were described as frequencies and compared between groups using the chi-squared or Fisher's exact test. The Wilcoxon test was used for paired analysis in order to detect significant variations in each intervention group.

## Results

**Figure 1:**
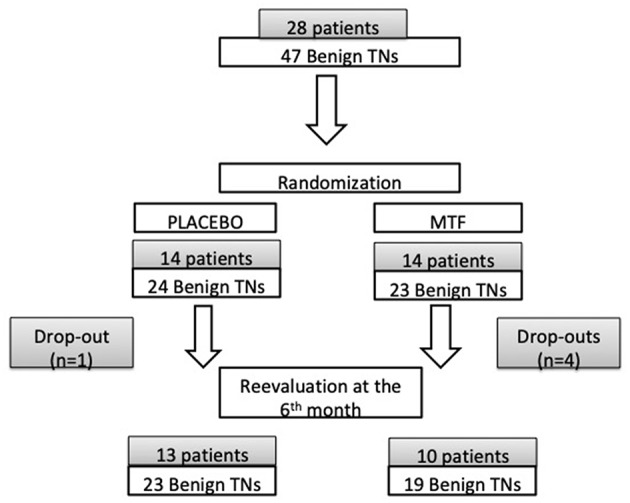
Flowchart of participants in the study.

Twenty-eight individuals with benign TNs agreed to participate in this prospective study. Most patients had more than one TN; therefore, a total of 47 TNs were initially evaluated at the beginning of the prospective study. Patients were randomized into two groups: (1) 14 patients (24 TNs) receiving placebo (P) and (2) 14 patients (23 TNs) receiving metformin (MTF). Five patients were excluded because of lack adherence to the protocol or for not attending the 6 month evaluation: 1 patient from the P group (1 TN) and 4 patients from MTF group (4 TNs). Figure 1 shows the flowchart of the study participants.

A total of 42 nodules were monitored for 6 months (23 in *P* group and 19 in MTF group). Comparisons of baseline characteristics between groups showed that the two groups were similar at the moment of randomization and maintained this similarity at the end of the study with respect to the studied variables of interest (Table 1). The median UIC also did not differ between groups and showed a high frequency of excessive or more than adequate iodine status among participants, according to the WHO criteria (21) as shown in Table 1. Finally, the majority did not have any metabolic syndrome according to National Cholesterol Education Program (NCEP) criteria (22) or IR according to the Brazilian Metabolic Syndrome Study (BRAMS) (20).

**Table 1:** Baseline characteristics of interesting variables between the studied groups.

	**Placebo (*n =* 13)**	**MTF (*n =* 10)**	P^*^
**EVALUATION BY PATIENTS THAT CONCLUDED THE STUDY**			
AGE (years)	53 (22–74)	50.5 (28–78)	0.98
FEMALE (%)	92.3	100	0.56
BMI (kg/m^2^)	26.7 (20.4–39.1)	30.0 (19.7–38.9)	0.56
TV (cm^2^)	14.2(4.2–60.7)	13.7 (4.5–38.4)	0.93
TSH (μU/mL)	1.1 (0.4–3.0)	1.5 (0.4–3.5)	0.15
HOMA IR	1.7 (1.0–6.0)	1.95 (0.8–5.9)	0.78
IR + (%)	23.1	20.0	0.63
HDL (mg/dL)	53 (33–86)	56 (47–83)	1.00
TG (mg/dL)	91.5 (25–174)	103 (46–130)	0.74
MS NCEP (%)	23.1	20.0	0.63
TPO-AB + (%)	8.3	11.1	0.69
UIC (μg/L)	183.4 (97.5–518)	203 (71–569)	1.00
Adequate UIC (%) #	41.7	40	0.64
Insufficient UIC (%) ##	8.3	10	0,71
More than adequate or excessive UIC (%) ###	50	50	0.66
**EVALUATION BY NODULES FROM PATIENTS THAT CONCLUDED**			
**THE STUDY**			
	**MTF (*****n=*** **19)**	***P*** **(*****n=*** **23)**	P^*^
TNV (cm^3^)	0.70 (0.074–16,93)	0.78 (0.031–25.3)	0.88

P

* *Mann-Whitney test comparing MTF and Placebo groups*.

*MTF, metformin; BTN, benign thyroid nodules; BMI, body mass index; TNV, thyroid nodule volume; TV, thyroid volume; TSH, thyroid-stimulating hormone; HOMA, homeostasis model assessment; IR+, insulin resistance present [defined by BRAMS (20)]; HDL, High Density Lipoprotein; TG, Triglycerides; MS NCEP, metabolic syndrome by National Cholesterol Education Program criteria (22); TPO-AB+, thyroperoxidase antibodies positive; UIC, urinary iodine concentration. According to WHO (21): # <100 μg/L; ## 100–199 μg/L; ### ≥ 200 μg/L*.

After 6 months, serum TSH was significantly reduced in patients using MTF (−0.21 vs. 0.09 mUI/L in the *P* group; *p* = 0.015) as shown in Table 2. This pattern of reduction of serum TSH with MTF also occurred after excluding those five patients with IR; however, a borderline statistical significance (−0.21 vs. 0.10; *p* = 0.10) as shown in Table 2 was noted. Considering the outcomes of the entire group, there were no statistically significant variations in TN volume, TV, HOMA-IR, or BMI in each intervention group. Moreover, there were no significant differences in the variations between groups. However, tendencies for enlargement of benign nodular goiter (BNG) with placebo (16% from baseline TV; *p* = 0.093) and for a protective effect of MTF on growing TNs (OR: 0.25; IC:0.05–1.20) when those patients with IR were excluded from analysis were detected as shown in Table 2.

**Table 2:** Comparisons between outcome variables in MTF and Placebo groups during the study.

	**Whole group**			After excluding those with IR^**^		
	**MTF (*n =* 10)**	**Placebo (*n =* 13)**	P^*^	**MTF (*n =* 8)**	**Placebo (*n =* 10)**	P^*^
**EVALUATION BY PATIENTS THAT CONCLUDED THE STUDY**						
ΔTSH (mUI/L)	**−0.21(0.61)^a^**	**0.09(0.24)**	**0.015**	−0.21 (0.6)^b^	+0.10(0.3)	0.100
ΔTV (cm^3^)	−0.36(4.36)	+2.55 (6.53)	0.313	−1.13(4.0)	+2.68(8.7)^b^	0.100
Δ%TV	−0.009 (0.45)	0.16 (0.45)	0.832	−0.04(0.4)	0.16 (0.4)	0.573
Thyroid volume that growth#	30%	53.8%	0.237	25%	60%	0.157
ΔHoma	−0.1(1.95)	−0.1 (0.85)	0.738	−0.10 (1.3)	0.00 (0.43)	0.762
ΔBMI (kg/m^2^)	+0.05 (1.4)	0.00 (0.86)	0.446	+0.15 (1.1)	+0.2 (1.57)	0.696
**EVALUATIONS BY NODULES FROM PATIENTS THAT CONCLUDED THE STUDY**						
	**MTF (*****n=*** **19)**	**Placebo (*****n=*** **23)**	P^*^	**MTF (*****n=*** **15)**	**Placebo (*****n=*** **18)**	P^*^
ΔBTN Volume (cm^3^)	0.000(0.44)	+0.006(0.52)	0.870	−0.05 (0.26)	+0.02 (0.76)	0.259
Δ%BTN Volume	0.00(0.31)	0.014(0.61)	0.695	−0.05 (0.4)	+0.076(0.6)	0.401
BTNs that growth #	31.6%	47.8%	0.227	20%	50%	0.070

a P = 0.037 (Wilcoxon test –paired analysis);

b *P = 0.09 (Wilcoxon test –paired analysis)*.

NE, not evaluated; P

* Mann-Whitney comparison between groups; IR, insulin resistance [

** *defined by BRAMS (20)]; MTF metformin; TSH, thyroid-stimulating hormone; TNV, thyroid nodule volume; TV, thyroid volume; BTNs, benign thyroid nodules; BMI, body mass index; #: growth of more than 10%. In paired analysis only p ≤ 0.10 are highlighted*.

In this subgroup of nodules (with IR), a lower median UIC was observed compared to the remainder of the group (139 [119] vs. 207 [206]; *p* < 0.01) in addition to a higher frequency of iodine insufficiency (33.3 vs. 6.7%; *p* = 0.07).

A stratified analysis by iodine status showed that the reduction in serum TSH levels with MTF was maintained in the population without iodine insufficiency (−0.24 vs. +0.07 in the P group; *p* = 0.046) and was accentuated in those with excessive or more than adequate UIC (−0.69; *p* = 0.043) as demonstrated in Table 3. A protective effect of MTF on growing TN was also suggested (OR: 0.11; IC: 0.02–0.84) in those with higher UIC.

**Table 3:** Stratified analysis by iodine status: comparisons between outcome variables in MTF and Placebo groups during the study.

	**Insufficient UIC (<100μg/L)**			**Adequate UIC (100–199** **μg/L)**			**More than adequate** **+** **excessive UIC (≥** **200μg/L)**		
	**MTF (*n =* 1)**	**Placebo (*n =* 1)**	P^*^	**MTF (*n =* 4)**	**Placebo (*n =* 5)**	P^*^	**MTF (*n =* 5)**	**Placebo (*n =* 6)**	P^*^
**EVALUATIONS BY PATIENTS THAT CONCLUDED THE STUDY BY IODINE STATUS**									
ΔTSH (mUI/L)	–	–	NE	−0.06 (0.64)	+0.07(0.32)	0.413	**−0.69 (0.41)***^a^*	+0.07(0.58)	0.082
ΔTV (cm^3^) Δ%TV	–	–	NE	+0.61(7.2) +0.22(0.56)	+2.8(8.46) +0.15(0.58)	0,730 1.0	−1.4 (2.97) −0.11(0.2)	+2.0 (8.9) +0.16(0.52)	0.082 0.247
Thyroid volume that growth^#^	–	–	NE	50%	60%	0.76	0%	50%	0.120
ΔHoma	–	–	NE	−0.30(1.43)	−0.10(1.55)	0.730	+0.2 (3.7)	0.0 (1.2)	0.792
ΔBMI (kg/m^2^)				−0.25(1.6)	0.00(2.35)	0.556	+0.2(1.8)	+0.05(1.7)	0.662
**EVALUATIONS BY NODULES FROM PATIENTS THAT CONCLUDED THE STUDY**									
	**MTF (*****n=*** **2)**	**Placebo (*****n=*** **3)**	P^*^	**MTF (*****n=*** **7)**	**Placebo (*****n=*** **7)**	P^*^	**MTF (*****n=*** **10)**	**Placebo (*****n=*** **10)**	P^*^
ΔBTN Volume (cm^3^)	+0.15 (–)	−0.006 (–)	1.00	+0.17(0.64)	+0.03(0.57)	0.535	−0.029 (0.28)	+0.10 (2.8)	0.075
Δ%BTN Volume	−0.062 (–)	−0.19 (–)	0.80	+0.06(0.58)	+0.01(0.62)	0.805	−0.03(0.32)	+0.24(0.5)	0.075
BTNs that growth ^#^	50%	33.3%	0.700	42.9%	42.9%	0.704	**20%**	**70%**	**0.035**

a P = 0.043 (Wilcoxon test- paired analysis);

* *Mann-Whitney test between MTF and Placebo group; NE: not evaluated; UIC: Urinary iodine Concentration; MTF metformin; TSH, thyroid-stimulating hormone; TNV, thyroid nodule volume; TV, thyroid volume; BTNs, benign thyroid nodules; BMI, body mass index*.

# *: growth of more than 10%. In paired analysis only p ≤ 0.10 are highlighted*.

The median baseline TSH in the groups with adequate, insufficient, or excessive/more than adequate iodine status were similar (1.5, 1.8, and 1.4 μU/L, respectively; *p* = 0.764).

## Discussion

In this study, 6 months of MTF therapy caused a reduction in TSH levels in euthyroid subjects without significant BMI reduction. These are significant data since it might be one of the diverse postulated mechanisms through which MTF could reduce thyroid and benign TN volumes. Some studies have already suggested that MTF therapy is associated with a reduction in TSH levels, but the majority of them evaluated patients with overt or subclinical hypothyroidism, and all of them evaluated only patients with diabetes or IR (7–10, 12–19). A meta-analysis, including seven studies in which changes in TSH levels in patients receiving MTF were evaluated, showed a reduction in TSH levels both in overt and in subclinical hypothyroidism but with no change in euthyroid patients (12). Moreover, in a double-blinded placebo-control clinical trial, MTF treatment was associated with a decrease in TSH levels only in patients with TSH >2.5 μU/mL (8). A preliminary study demonstrated that MTF administration in subjects with IR was associated with significant reduction in TSH levels and increase in levels of free tri-iodothyronine (FT3) with no change in free tetra-iodothyronine (FT4) (7). In a study with elderly people with type 2 diabetes, the patients with diabetes who were given MTF had lower TSH levels compared to non-diabetic patients in addition to patients treated with other antidiabetic agents (13).

Mechanisms by which MTF lowers TSH levels are still being debated. Some hypotheses mention a change in the affinity and/or quantity of thyroid receptors, an increase in central dopaminergic tone, or a direct effect on TSH regulation, thereby enhancing the effects of thyroid hormones in the pituitary gland (14, 15). The central effects of metformin on TRH/TSH regulation might involve the AMPK system. MTF decreases hepatic gluconeogenesis via AMPK activation; otherwise, its activity is inhibited in the hypothalamus and possibly enhances the inhibition of thyroid hormones on the pituitary gland (12).

It is well-known that TSH and insulin are involved in thyrocyte proliferation, and several studies have reported that patients with insulin resistance might have large thyroid volumes and a higher prevalence of TNs (2, 3). Therefore, the reduction of TSH and HOMA-IR after MTF treatment could be causative factors for TN volume reduction. However, contrary to previous studies, the current work revealed no impact of MTF on HOMA-IR. The principal hypothesis that has been suggested to explain these results is the small selected sample, which consisted of predominantly patients without IR. The low levels of baseline HOMA-IR make it difficult to find a significant decrease in this index throughout the study period. In summary, the effect of variations on this index caused by MTF might be weakened. Reanalyzing data after excluding patients with IR, an increase in BNG volume with placebo was detected; however with a borderline *p*-value. The evaluation of this small group with IR showed an association with iodine insufficiency that might blunt BNG volume response to MTF.

Iodine deficiency is a well-known risk factor for nodular thyroid disease. Additionally, lower UIC has been found among patients with diabetes and obesity compared to control subjects, and urinary iodine was shown to be negatively correlated with glucose, insulin levels, and HOMA-IR (23–26). Therefore, hypothesizing that iodine status could characterize a selection bias and could exert some influence on the effect of MTF on thyroid; we assessed UIC from the participants in our study. This was a differential approach, in comparison to studies from other researchers. Rezzónico et al. (6) and Karimifar et al. (8) did not assess UIC in their study; however, they reported previous studies that involved that participants residing in an iodine-sufficient area. Anil et al. (7) did not report UIC in their study participants. The median of UIC indicates that our sample did not have an insufficient or excessive iodine status according to WHO criteria (21); however, a high frequency of excessive/more than adequate UIC was found in accordance with previously reported studies from the same country (27, 28).

In the present study, the stratified analysis, by iodine status, showed that the MTF effects were more evident in the subgroup with higher UIC. We speculate that in this sample the reduction of serum TSH by MTF was important for preventing BNG growth since excessive UIC has been associated with higher levels of serum TSH and subclinical hypothyroidism in previous studies from the same region (28, 29). We could not prove this hypothesis since we did not demonstrate different TSH levels according to iodine status. Other possible hypothesis might be related to pro-inflammatory factors that were not measured in the present study but could be potentially associated with excessive iodine status; several studies have reported that high iodine intake is one of environmental factors implied in Hashimoto thyroiditis pathogenesis (30, 31).

MTF has diverse antitumor effects not solely related to reduction of insulin resistance. The insulin/insulin-like growth factor 1 (IGF-1) signaling pathway has long been known to promote cell proliferation and decrease apoptosis (32). Previous studies have shown the potential inhibitory effect of metformin on the growth of human thyroid cells (11). The potential antitumor effect of MTF is complex and multifactorial. Two distinct but not exclusive mechanisms can be implicated in this action. First, by decreasing insulinemia, MTF can exert an inhibitory effect via insulin/IGF-1 signaling (11). Second, MTF might also directly inhibit cell growth by multiple molecular pathways, including AMPK-dependent (mTOR) and AMPK-independent mechanisms (11, 33, 34). Other possible actions include an effect of MTF on tumor stem cells and the nuclear factor κB (NF-κB) pathway (33). Furthermore, a recent study reported that MTF could inhibit the secretion of CXCL8 stimulated by tumor necrosis factor-α (TNF-α) in primary cultures of normal thyroid cells and differentiated thyroid cancer cells (35).

Some studies have demonstrated that MTF, an insulin sensitizer, could help reduce TN volume. Rezzónico et al. (6) reported the effect of MTF on reduction in nodular size in patients with small benign TN and IR after 6 months of follow-up. Recently, two meta-analysis demonstrated a significant decrease in TN volume after MTF therapy by pooling the results of smaller studies (9, 10). Miao et al. (9) included seven studies with a total of 240 subjects and He et al. (10) included five studies with a total of 189 subjects. The time of follow-up in these studies ranged from 3 to 12 months. Anil et al. found that MTF therapy caused a significant decrease in TV and nodule size in patients with IR (7) although, the population of the study had obesity and had a significantly decreased BMI after diet and exercise was prescribed. Diet and exercise could have contributed to the reduction in HOMA-IR and thyroid and nodule volumes.

The present study has certain limitations. Our sample was small, which might have limited the statistical power of the analysis; however, this was also a limitation for other randomized controlled trials included in the meta-analysis (9, 10). Rezzónico et al. (6) evaluated 14 patients in MTF group (19 TNs), and Karimifar et al. (8) included 43 subjects in the MTF group (35 TNs). Our sample also had a few patients with IR, but to the best of our knowledge, it is the first time that the effect of MTF in TSH and TN volume was evaluated in patients without IR. Free thyroxine (FT4) was not assessed to evaluate if the reduction in TSH was accompanied by an increase in FT4. The follow-up time (6 months) might have been insufficient to observe a reduction in nodular goiter size; however, the follow-up period in the majority of studies included in the meta-analysis was 6 months. Only one study had a 1 year follow-up time (9, 10).

In conclusion, this study demonstrated that MTF caused a reduction in TSH levels in patients with BNG. This effect was more accentuated in patients with higher levels of UIC and was accompanied by a suggested protective effect on TN enlargement. Future studies with larger samples are necessary in order to evaluate the effect of MTF in benign thyroid nodules in a more detailed and conclusive manner.

## Data Availability

The datasets generated for this study are available on request to the corresponding author.

## Ethics Statement

This study was carried out in accordance with the recommendations of Ethical Committe of Clementino Fraga Filho University Hospital with written informed consent from all subjects. All subjects gave written informed consent in accordance with the Declaration of Helsinki. The protocol was approved by the Ethical Committe of Clementino Fraga Filho University Hospital (CAAE:35585214.0.0000.5257).

## Author Contributions

PdS, PT, FC, MV, BdA, and DdC designed the study and discussed the results as well as providing contributions on writing and revising the manuscript. PT and PdS participated on final submission of the paper. BdA and LG organized the database. PdS, PT, and LG conducted the clinical trial and evaluated all patients during the study, collecting data, performing the ultrasounds and assessing laboratory data. PT and PdS analyzed and wrote the results.

### Conflict of Interest Statement

The authors declare that the research was conducted in the absence of any commercial or financial relationships that could be construed as a potential conflict of interest.

## Abbreviations

IR: insulin resistance
MTF: metformin
TN: thyroid nodule
TSH: thyroid stimulating hormone
TV: thyroid volume
AMPK: adenosine monophosphate-activated protein kinase
mTOR, mammalian target of rapamycin BTN: benign thyroid volume
BMI: body mass index
TPO-Ab: thyroid peroxidase antibody
UIC: Urinary Iodine Concentration
P: placebo
WHO: World Health Organization
BNG: benign nodular goiter
NF-κB: nuclear factor κB
TNF-α: tumor necrosis factor-α
FT4: tetra-iodothyronine.
